# iOPTICS-GSO for identifying protein complexes from dynamic PPI networks

**DOI:** 10.1186/s12920-017-0314-x

**Published:** 2017-12-28

**Authors:** Xiujuan Lei, Huan Li, Aidong Zhang, Fang-Xiang Wu

**Affiliations:** 1 School of Computer Science, Shaanxi Normal University, Xi’an, Shaanxi China; 2 Department of Computer Science and Engineering, State University of New York at Buffalo, NY, 14260-2000 USA; 3 School of Mathematical Sciences, Nankai University, Tianjin, China; 4 Division of Biomedical Engineering and Department of Mechanical Engineering, University of Saskatchewan, Saskatoon, Canada

**Keywords:** Ordering points to identify the clustering structure algorithm (OPTICS), Glowworm swarm optimization algorithm (GSO), Protein complex, Density-based clustering

## Abstract

**Background:**

Identifying protein complexes plays an important role for understanding cellular organization and functional mechanisms. As plenty of evidences have indicated that dense sub-networks in dynamic protein-protein interaction network (DPIN) usually correspond to protein complexes, identifying protein complexes is formulated as density-based clustering.

**Methods:**

In this paper, a new approach named iOPTICS-GSO is developed, which is the improved Ordering Points to Identify the Clustering Structure (OPTICS) algorithm with Glowworm swarm optimization algorithm (GSO) to optimize the parameters in OPTICS when finding dense sub-networks. In our iOPTICS-GSO, the concept of core node is redefined and the Euclidean distance in OPTICS is replaced with the improved similarity between the nodes in the PPI network according to their interaction strength, and dense sub-networks are considered as protein complexes.

**Results:**

The experiment results have shown that our iOPTICS-GSO outperforms of algorithms such as DBSCAN, CFinder, MCODE, CMC, COACH, ClusterOne MCL and OPTICS_PSO in terms of *f-measure* and *p-value* on four DPINs, which are from the DIP, Krogan, MIPS and Gavin datasets. In addition, our predicted protein complexes have a small *p-value* and thus are highly likely to be true protein complexes.

**Conclusion:**

The proposed iOPTICS-GSO gains optimal clustering results by adopting GSO algorithm to optimize the parameters in OPTICS, and the result on four datasets shows superior performance. What’s more, the results provided clues for biologists to verify and find new protein complexes.

## Background

Proteins are the indispensable components in various types of cells and tissues, and the executors of the biological functions. At the same time, each protein in the cell does not exist in isolation, and the occurrence of every life process must involve more than one protein [1]. Protein complexes are not only the basis of normal biological processes, also play important role in the pathological processes [2]. Therefore, identifying protein complexes play an important role in understanding the cellular organizations and functional mechanisms [3]. As a variety of protein interaction database have produced, it is possible to identify protein complexes from protein-protein interaction (PPI) networks. Living organisms are always changing, so are PPIs in living cells [4]. In addition, the interactions between proteins are changing over time not only with the presence and degradation of protein, but also with the environment. In [5], the authors incorporated the “time” factor for proteins in the form of cell-cycle phases into the analysis of complexes and studied the dynamic phenomena of complexes assembly and disassembly across various cell cycles. To express the dynamics, many dynamic data, including gene expression profiles [6], have been used to construct dynamic PPI networks (DPINs).

The discovery of protein complexes is equivalent to find subsets of function-related proteins from a data set. Clustering is an effective method, which can find subsets that have some common attributes from the database [7]. Therefore, the development of improved clustering algorithms has received a lot of attention in the last few years. The clustering algorithm based on density is an important type of clustering analysis method and one of its main advantages is able to detect any shape of cluster while being not sensitive to noise [8]. The Density-Based Spatial Clustering of Applications with Noise (DBSCAN) [9], which was proposed by Ester et al., is a clustering algorithm based on density. The DBSCAN algorithm is applicable to any shape and size of the dataset. It is noise-tolerant and independent of ordering of data objects. However, it has two initial parameters, the field radius and the minimum point within the field radius. The DBSCAN algorithm requires the user to manually input these two parameters while the clustering results are very sensitive to the values of two parameters. The DBSCAN algorithm also needs initialization parameters. In order to overcome those shortcomings of DBSCAN algorithm, Ankerst et al. [10] proposed a new algorithm called Ordering Points to Identify the Clustering Structure (OPTICS). Its basic idea is similar to DBSCAN when identifying clusters, and both searching for high density regions.

In real life, many optimization problems require not only to calculate the extremum, but also obtain their optimal values. This kind of problem is a serious challenge to the traditional algorithm. In this case, a growing number of swarm intelligence algorithms are successively put forward, such as Genetic Algorithm (GA) [11], Particle Swarm Optimization (PSO) [12]. Glowworm swarm optimization algorithm (GSO) [13], proposed by Krishnan and Ghose in 2005, is a bionic swarm intelligence algorithm. GSO simulates the glowworm group in motion guided by fluorescence to attract other glowworms or foraging around, the greater the value of fluorescein, the bright the glowworm is, and the more attractive it is.

OPTICS algorithm does not produce cluster for a data set explicitly; but instead creates an augmented ordering queue representing its density-based clustering structure. Then we need to deal with cluster-ordering and get clustering results. For each network clustering, different parameters settings produce different results. In this study, we put forward the algorithm named iOPTICS-GSO which is the improved OPTICS algorithm by using GSO to optimize the parameters in OPTICS. In order to investigate its performance, iOPTICS-GSO with other seven computing methods including DBSCAN [9], CFinder [14], MCODE [15], CMC [16], COACH [17], ClusterOne [18], MCL [19] and OPTICS_PSO [20]. At the same time, we also use the *p-value* for function enrichment analysis. The experiment results illustrated that iOPTICS-GSO achieved better performance compared with other competing algorithms.

The outline of this paper is as follows. In Section 2, after reviewing the GSO algorithm, basic OPTICS and our iOPTICS-GSO are presented. In Section 3, experimental results and analysis are described and discussed, and the conclusions are in Section 4.

## Methods

### GSO algorithm

In the GSO algorithm, glowworms with higher fluorescein are more attractive to other glowworms, and thus a group of glowworms move towards the glowworms with high fluorescein. Each glowworm in its dynamic decision domain radius chooses a glowworm whose fluorescein value is higher than its own fluorescein value to move towards and updates its dynamic decision-making domain. Then some glowworms are selected according to probability to update the position from dynamic decision-making domain. Finally, the decision domain updated. GSO algorithm has two important phases as follows.

### The phase for updating the fluorescein.

The fluorescein value of each glowworm is related to the value of previous generation of fluorescein and the current fitness function. Let x_i_ (t) represent the location of the *i*-th glowworm in the *t*-th generation, *J*(x_i_(t)) represent the fitness function of the *i*-th glowworm in the *t*-th generation. The fluorescein value *l*_i_(t) of the i-th glowworm in the *t*-th generation is calculated as follows:

1 $$ {l}_i(t)=\left(1-\rho \right)\left({l}_i\left(t-1\right)\right)+\gamma J\left({x}_i(t)\right) $$

where *ρ* and *γ* are two parameters with the values between 0 and 1.

### The phase of updating the position.

Each new position of the glowworms is a small movement from the original position, which is calculated as follows:

2 $$ {x}_i\left(t+1\right)={x}_i(t)+s\times \left(\frac{x_j(t)-{x}_i(t)}{\left\Vert {x}_j(t)-{x}_i(t)\right\Vert}\right) $$

3 $$ s=\left(\frac{t_{\mathrm{max}}-t}{t_{\mathrm{max}}}\right)\times {s}_0 $$

where *S* is the update step length of the glowworms, S_0_ is the initial step length, and t_max_ is the largest number of iterations. Here, we adopt the method of linear regressive instead of fixed step length [21], in order to improve optimization ability of the algorithm when updating the population.

In the GSO, each glowworm is looking for the neighborhood within its field of vision, and then moves to a brighter glowworm. Each time the moving direction depends on the neighborhood selection. In addition, the glowworm decision domain radius size is influenced by the number of glowworms in different neighborhoods, when the number of glowworms is too small, glowworms will increase their decisions radius in order to find more glowworms; On the contrary, they will reduce their own decision-making radius. At the end, the GSO makes most of the glowworms gathered in a better position.

### Optics

The key idea of density-based clustering such as OPTICS is that for each object in a cluster the neighborhood within a given radius has to contain at least a minimum number of objects (*MinPts*), which is the cardinality of the neighborhood. The condition *Card*(*N*_*ε*_(*q*)) ≥ *MinPts* is called the “core object condition”. If this condition holds for an object *p*, then we call *p* a “core object”. Only from core objects, can other objects be directly density-reachable.

In PPI networks, the node degrees obey power-law distribution, we select all nodes as core nodes so that the node which degree is small can be considered. As a result, we redefined two definitions as follows.

**Definition 1:** (Distance_*core*_ of node *p*).

Let *p* be a protein in a PPI network, Distance_*MinPts*_ (p) be the *MinPts*-th maximum distance from node p to all the other nodes. Then, the core-distance of *p* is defined as follows:

4 $$ {\mathrm{Distance}}_{\mathrm{core}}\left(\mathrm{p}\right)={\mathrm{Distance}}_{\mathrm{MinPts}}\left(\mathrm{p}\right) $$

**Definition 2:** (Distance_reachability_ of node *p*).

Let nodes *p* and *o* be two proteins in a PPI network, let N(*o*) be the set which contains neighbors of node *o*. Then, the Distance_reachability_ is defined as follows:

5 $$ {\mathrm{Distance}}_{\mathrm{reachibility}}\left(\mathrm{p},\mathrm{o}\right)=\kern0.48em \max \left({\mathrm{Distance}}_{\mathrm{core}}\left(\mathrm{o}\right),{\mathrm{d}}_{\mathrm{op}}\right) $$

where d_op_ is the distance from node p to node o. As can be seen above, the reachability distance of a node cannot be smaller than the core distance of node *o*. Thus OPTICS creates an ordering queue of all nodes, and stores the core distance as well as a suitable reachability distance for each node.

### The proposed iOPTICS-GSO

In this section, we elaborate the proposed iOPTICS-GSO how to identify protein complexes. The following four subsections describe the calculation of distance between proteins, clustering PPI networks, iOPTICS-GSO algorithm and its time complexity analysis, respectively.

1. Calculating the distance in a PPI network

In a PPI network, we use the similarity between two proteins to measure their distance. As we know, the fewer the number of same neighbors between two proteins is, the less the similarity of two proteins is, and the smaller the probability that they belong to the same protein complex is. On the contrary, the higher the similarity of the two proteins is, the more likely they belong to the same protein complex [22]. Therefore, the similarity is determined according to the number of same neighbors the two nodes share in the PPI network. Consider a PPI network PN, A is adjacency matrix of PN, and the binary vector *X*_*i*_ = (A_*i1*_, *A*_*i2*_, …, *A*_*in*_) indicates the interactions between protein *i* and other proteins, then we calculate the number of common neighbor(CN) between proteins *i* and *j* by the equation: *CN*_*ij*_ = |*N*_*i*_∩*N*_*j*_|. Here *N*_*i*_ and *N*_*j*_ expresses the neighbor that proteins *i* and *j* have, respectively. Therefore, if CN_ij_ ≠ 0, the similarity between proteins *i* and *j* is calculated as follows [23]:

6 $$ {sim}_{ij}=\frac{\sum_{\mathrm{k}=1}^{\mathrm{n}}\min \left({\mathrm{CN}}_{\mathrm{ik}},{\mathrm{CN}}_{\mathrm{jk}}\right)}{\sum_{\mathrm{k}=1}^{\mathrm{n}}\max \left({\mathrm{CN}}_{\mathrm{ik}},{\mathrm{CN}}_{\mathrm{jk}}\right)} $$

Considering in the PPI network, the two nodes which have no common neighbor also have connection, and there have multiple protein complexes which only contains two proteins in standard complexes. we redefined the similarity *S* as follows:

7 $$ {S}_{ij}=\left\{{}_{{}_{\frac{\mathrm{Aij}}{\max \left(\left|{N}_i\right|,\kern1em \left|{N}_j\right|\right)},}\kern4.199999em {\mathrm{CN}}_{\mathrm{ij}=0.}}^{{\mathrm{sim}}_{\mathrm{ij}},\kern7.859997em {\mathrm{CN}}_{\mathrm{ij}}\ne 0;}\kern1.08em \right. $$

The greater the similarity between two proteins, the smaller the distance between them is. Then the distance can be calculated as follows:

8 $$ {\mathrm{D}}_{\mathrm{ij}}=1-{\mathrm{S}}_{\mathrm{ij}} $$

We use the *D*_*ij*_ to replace the Euclidean distance in OPTICS for measuring the distance between two proteins in a PPI network.

2. Clustering PPI network.

**Fig. 1:**
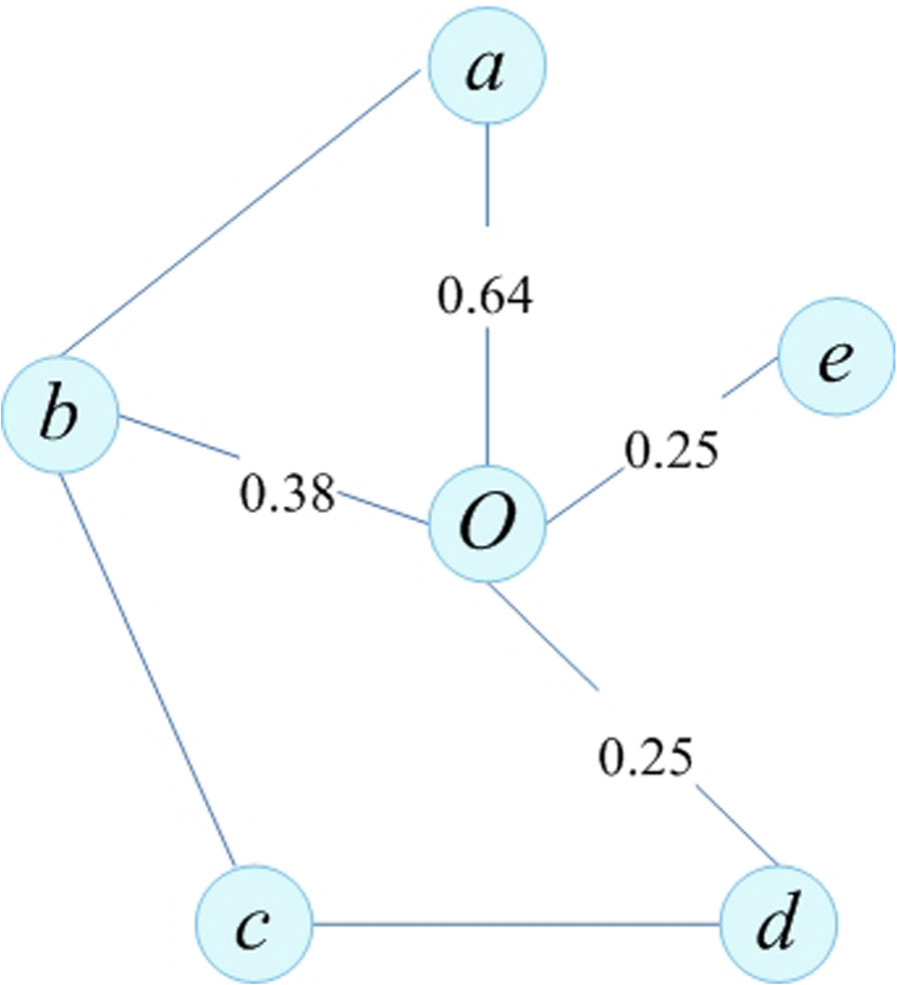
An example of the two distances

Fig. 1 shows a PPI network with distances between node o and other nodes. In this study, we set the *MinPts* to be 4, and then from Fig. 1, we select firstly the core to be node *o*. For obtaining the core distance of *o*, we calculate all distances between core *o* and its neighbors according to Eq. (8). From the definition, we get the value Distance_reachability_ (d, o) = 0.64. In the same manner, we obtain a sequence of values of all nodes.

**Fig. 2:**
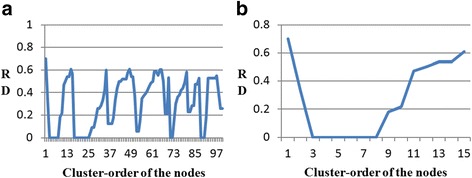
Illustration of the cluster-ordering (**a**) Reachability-plots for a part of DIP data by OPTICS (**b**) One cluster in Reachability-plots

We can now improve the algorithm to preserve the track of all the reachability distance values and use them to save the expensive operations identified above. We can obtain an augmented ordering queue from OPTICS, and convert the ordering queue into a reachability-plot. Fig. 2 shows such a reachability-plot and an example of cluster. Each sunken part in Fig. 2a can be viewed as a cluster. That is, the new cluster starts from a steep down region and end up with next steep down region. As a result, form the reachability-plot, the algorithm can find all clusters.

For example, in Fig. 2b we can see a cluster starting at object #1 and ending at object #15. Note that object #1, which is the last object with a high reachability value, is part of the cluster, its high reachability indicates that it is far away from the previous cluster. It has to be close to object #2. However, because object #3 has a low reachability value, indicating that it is close to one of the objects #1 or #2. Because the next object that OPTICS chooses is in the cluster-ordering, it has to be close to #2 (if it were close to object #l it would have been assigned index 1 and not index 2). A similar argument holds for object #15, which is the last object with a low reachability value, and therefore is also a member of the cluster.

3. iOPTICS-GSO Algorithm.

Although the OPTICS algorithm can find all clusters, the dynamic PPI network has more than one sub-network, and the size and topological structure of these sub-networks are quite different. For example, when we apply OPTICS to dynamic PPI network with 12 sub-networks, 12 reachability-plots are obtained; and each reachability-plot is different from others. The optimal parameters and the corresponding performance of each sub-network are shown in Table 1. It is evident that each sub-network has its own optimal parameters and the performances of the clustering result are different. It also can be seen that the OPTICS with global density parameters is not suitable for datasets with different densities.

**Table 1:** The value of parameters which corresponding to the best result in each sub-network on DIP

Timestamps	1	2	3	4	5	6	7	8	9	10	11	12
ɛ	0.62	0.50	0.52	0.59	0.60	0.58	0.62	0.51	0.55	0.64	0.60	0.60
MinPts	3	3	3	3	3	3	3	3	3	3	3	3
precision	0.7500	0.7421	0.8182	0.9000	0.8462	0.8889	0.9048	0.7805	0.6064	0.7419	0.6970	0.9524
recall	0.5263	0.5122	0.3971	0.4500	0.4400	0.2587	0.3115	0.5565	0.5089	0.5897	0.5349	0.5882
f-measure	0.6185	0.6000	0.5347	0.6000	0.5789	0.4324	0.4634	0.6497	0.5534	0.6571	0.6053	0.7273

It is well known that the GSO algorithm has less parameters, simple operation and good stability, etc. GSO algorithm simulates the characteristic of glowworms glow in nature, by comparing the size of the fluorescein value to achieve the purpose of communication, so as to realize the optimization of the problem. So we introduce the GSO algorithm to optimize the parameters of OPTICS, in order to obtain optimal results. Algorithm2 describes the details of iOPTICS-GSO. After several circulations iterative process, a glowworm constantly updates its position and iteratively approaches to the best position. At last, the glowworm finds the best position.

**Fig. 3:**
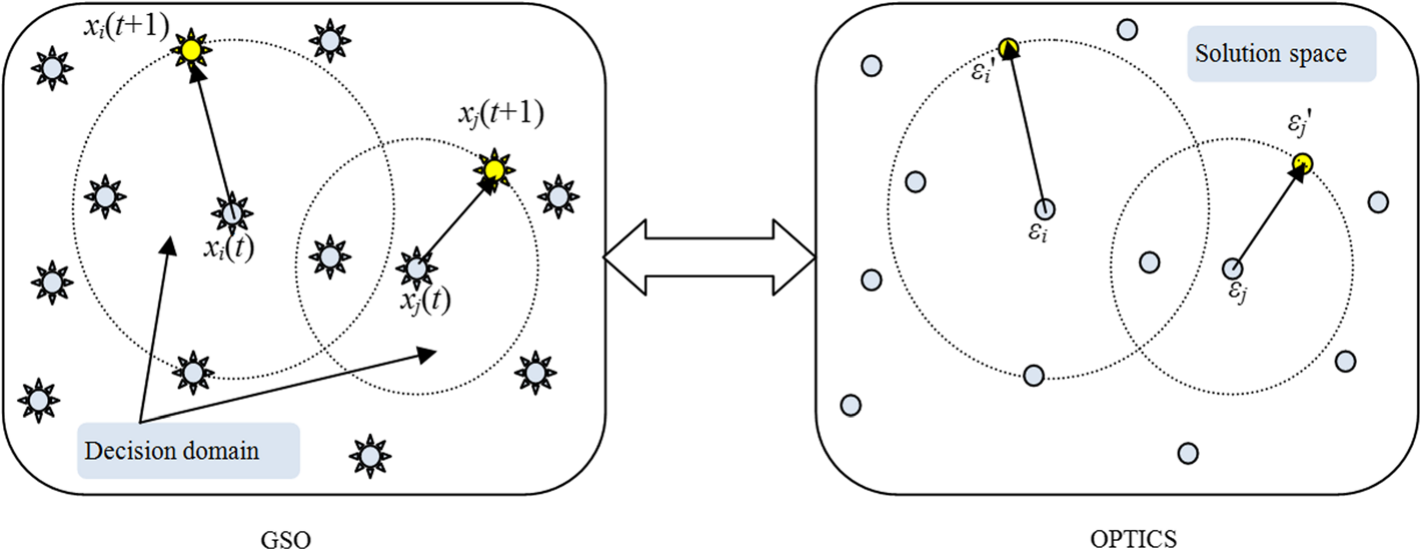
The corresponding relationships between GSO and OPTICS algorithms

The corresponding relationships between GSO and OPTICS are showed in Fig. 3. When we adopt the GSO algorithm to optimize the parameter ɛ in OPTICS, the position of glowworms in GSO also is related to the value of parameter ɛ. By updating its dynamic decision domain radius, a glowworm moving its position corresponds to searching for the optimal value of parameters ε. When fitness function achieves the maximum value in GSO after a number of positions are updated, OPTICS finds the best clustering result.

In Algorithm: iOPTICS-GSO, firstly, the fluorescein values, the decision domain radius and the positions of glowworms are initialized. Secondly, GSO algorithm is used to optimize the parameter ɛ in OPTICS. In this part, one position of a glowworm is one parameter value. Then OPTICS is run by using this parameter value. For each value (position), a corresponding clustering result is obtained. Next the clustering performance is evaluated for each value (position). Next the fluorescein value is updated and the glowworms move accordingly. After iterations, the new positions of glowworms are found. The maximum fitnessvalue is selected as the optimal position.

4. Time complexity analysis of iOPTICS-GSO algorithm

The time complexity is used to estimate the efficiency of the iOPTICS-GSO algorithm. Let *maxiter* be the maximal iterations of external loop in iOPTICS-GSO algorithm, *num* be the number of proteins in sub-works and *PopSize* be the number of glowworms. The time complexity is analyzed below:

- The time complexity of OPTICS algorithm is *O* (*num* ^2^).
- The time complexity of computing the fitness of glowworms is *O* (*PopSize* * *O* (*num* ^2^).
- The time complexity of glowworms moving process is *O* (*PopSize* ^2^).
- The time complexity for updating the position *O* is (*PopSize*).

In summary, the time complexity of iOPTICS-GSO is *O* (*maxiter* * (*num* ^2^ + *PopSize* * *num* ^2^+ *PopSize* ^2^ + *PopSize*)). Finally, the time complexity of this algorithm is *O* (*maxiter* * *PopSize* * *num* ^2^).
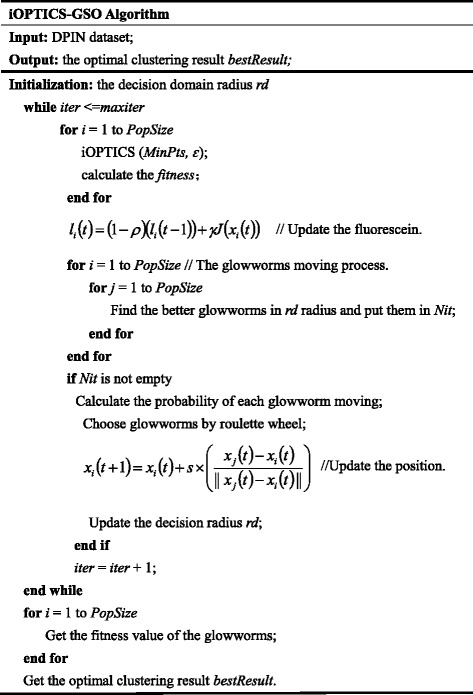


## Results and discussion

### Experimental datasets

In this study, we used four static PPI networks for yeast, including DIP [24], Krogan [25], MIPS [26] and Gavin [27] to evaluate our proposed iOPTICS-GSO. The DIP data consists of 4995 proteins and 21,554 interactions, Krogan data consists of 2674 proteins and 7075 interactions, MIPS data consists of 4546 proteins and 12,319 interactions and Gavin data consists of 1430 proteins and 6531 interactions. For verifying protein complexes identified by our proposed method, the set of protein complexes derived from CYC2008 [28] is selected as the gold standard dataset in this study, which includes 408 protein complexes and covers 1492 proteins,

In study, we construct DPINs similar to Ref. [29] by integrating gene expression profiles. Gene expression data were available from GEO (Gene Expression Omnibus) [30] with access number GSE3431. The data contained 9336 genes at 36 time points in the 3 cell life cycles. DPINs are constructed from static PPI network and gene expression data, we use the three-sigma principle to judge whether a gene is expressed in a particular timestamp. For example, we preset a threshold value, if the value of a protein is greater than the threshold at a certain timestamp t, this protein is judged to be an active protein at t timestamp. Each sub-network is constituted by these active proteins and the interactions between them. Then these sub-networks together form the DPIN. As a result, we get four DPINs from DIP, Krogan, MIPS and Gavin, respectively. Table 2 shows different scales of different sub-networks from these four static PPI networks.

**Table 2:** The number of proteins and interactions in each sub-network of the four datasets contain

DIP data	Timestamps	1	2	3	4	5	6	7	8	9	10	11	12
	Proteins	797	941	796	623	610	530	493	944	1090	591	661	461
	Interactions	981	1444	1188	745	750	646	573	1705	2185	856	974	526
Krogan data	Timestamps	1	2	3	4	5	6	7	8	9	10	11	12
	Proteins	336	379	320	256	206	189	202	580	626	304	330	250
	Interactions	334	464	331	234	210	184	213	1025	1081	314	373	258
MIPS data	Timestamps	1	2	3	4	5	6	7	8	9	10	11	12
	Proteins	737	897	781	583	570	531	470	839	1,014	523	616	402
	Interactions	1097	1443	1183	754	684	642	504	1238	1637	878	1207	700
Gavin data	Timestamps	1	2	3	4	5	6	7	8	9	10	11	12
	Proteins	177	228	215	135	112	102	96	379	419	174	190	146
	Interactions	242	334	317	150	135	118	135	1019	1043	230	264	184

### Performance evaluation

In order to evaluate the clustering results, we have adopted three kinds of commonly used statistical metrics: *precision*, *recall* and *f-measure* [31]. Precision and recall measure the accuracy of the protein complexes identified by algorithm matching the known protein complexes in the standard dataset and the accuracy of the known protein complexes matching the identified protein complexes, respectively. *f-measure* is used to evaluate the closeness between the known protein complexes and the identified protein complexes. *Precision*, *recall* and *f-me*asure are calculated as follows:

9 $$ precision=\frac{\left|X\cap F\right|}{X} $$

10 $$ recall=\frac{\left|X\cap F\right|}{\left|F\right|} $$

11 $$ f- measure=\frac{2\times \left( precision\times recall\right)}{precision+ recall} $$

12 $$ OS\left( pc, kc\right)=\frac{{\left| pc\cap kc\right|}^2}{\left| pc\right|\times \left| kc\right|} $$

where *X* is the set of proteins in an identified protein complexes and *F* is the set of known complexes in the standard dataset. |*pc*| is the number of proteins in the identified protein complex and |*kc*| is number of proteins in the known protein complex. The overlapping score (OS) evaluates how many proteins in the true protein complexes can be recovered by the identified protein complexes [32, 33]. Usually we consider an identified protein complex matches the known protein complex when the OS is equal to or larger than 0.2 [5]. We also use the *p-value* to evaluate the statistical and biological significance of the identified protein complexes [34]. In detail, given *k* proteins in a true protein complex *C* with a biological function shared by an identified proteins complex F from a total set *V* of proteins, the *p-value* is defined as:

13 $$ P- value=1-\sum \limits_{i=0}^{k-1}\frac{\left(\begin{array}{c}\left|F\right|\\ {}i\end{array}\right)\left(\begin{array}{c}\left|V\right|-\left|F\right|\\ {}\left|C\right|-i\end{array}\right)}{\left(\begin{array}{c}\left|V\right|\\ {}\left|C\right|\end{array}\right)} $$

which is the probability that an identified protein complex is enriched by a true protein complex only by chance [35]. A low *p-value* of an identified protein complex means the collective occurrence of these proteins belongs to the same complex not by chance, yet with a high statistical significance. That is to say, the lower the *p-value* of a protein complex is, the stronger biological significance the protein complex possesses, while the protein complex with *p-value* greater than 0.01 is considered to be insignificant. In the experiments, *p-value* was calculated on biological process ontologies.

### The effect of parameter

**Fig. 4:**
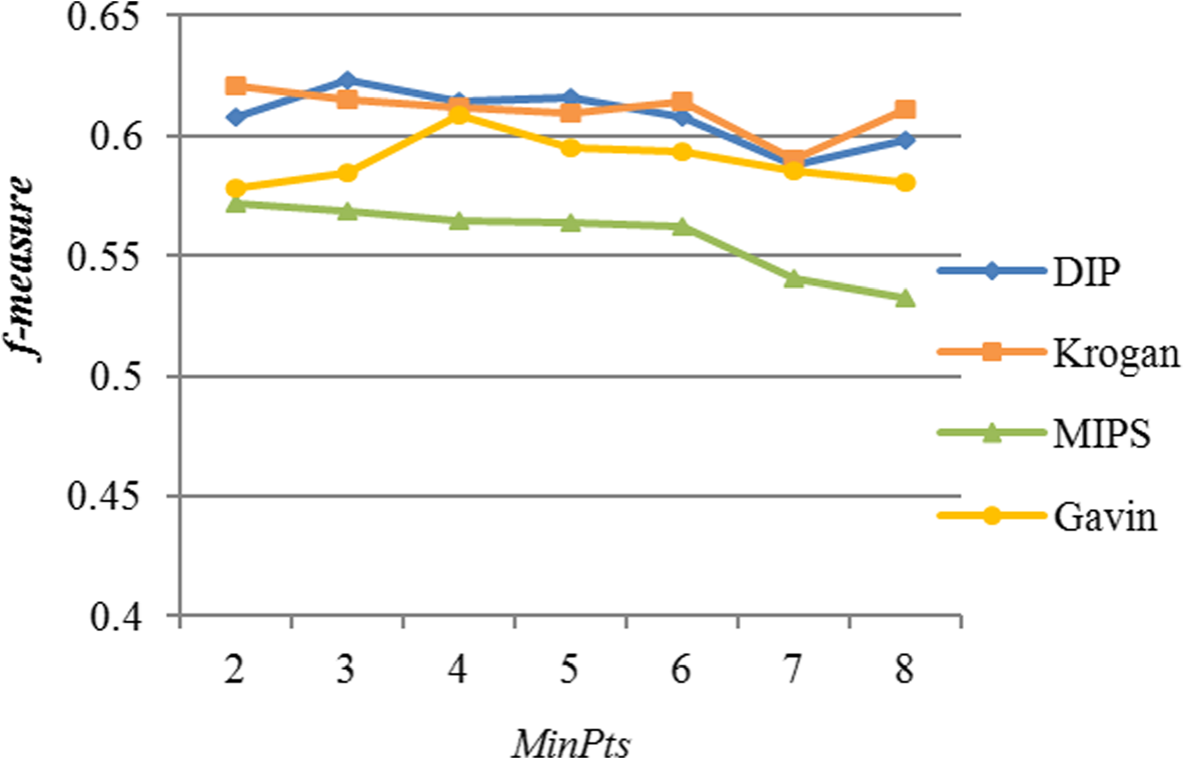
The effect of different values of MinPts on f-measure

In iOPTICS-GSO algorithm, there is one parameter to be preset, which is the value of *MinPts*. According to the topological properties of PPI networks, if the value of *MinPts* is too large, there would be no meaningful cluster that can be identified by the algorithm. For example, when we set *MinPts* to 10, there is no meaningful cluster that can be identified from the DPIN network. On the contrary, if the value of *MinPts* is too small, it will be too many proteins in the same cluster and the number of identified protein complexes will be few. In this study, the value of *MinPts* is set according to Fig. 4 for the four datasets. The x-axis represents the values of parameter which range from 2 to 8, and the y-axis represents the values of *f-measur*e. Each value of parameter corresponds to a value of *f-measure,*a set of values form the line chart, as shown in Fig. 4. The blue line represents the result on DIP data, the orange line represents the result on Krogan data, the green line represents the result on MIPS data, and the yellow line represents the result on Gavin data.

In Fig. 4, the effect of different values of *MinPts* on *f-measure* is not very big, and this also confirms that the reachability-plot is rather insensitive to the input parameter of the method. We observe that the value of *f-measure* increases initially as the value of *MinPts* increases and decreases after reaching the maximum. Then we chose the value of *MinPts* at which the f-measure reaches the maximum in iOPTICS-GSO. As a result, we find that the optimal values of *MinPts* are 3, 2, 2 and 4 for DIP, Krogan, MIPS and Gavin, respectively.

### Clustering comparisons

In order to directly validate its performance, the iOPTICS-GSO is compared with other seven competing algorithms, DBSCAN [9], CFinder [14], MCODE [15], CMC [16], COACH [17], ClusterOne [18] MCL [19] and OPTICS_PSO [20]. At the same time, the iOPTICS-GSO is also compared with the basic OPTICS. All comparisons are on the DIP, Krogan, MIPS and Gavin datasets. Each algorithm uses its best parameter when comparing, and it was found that these algorithms can get best results under the default parameter setting. The performances of all clustering algorithms are reported in Table 3 which contains the category of each algorithm, the number of identified protein complexes, and the average size of protein complexes.

**Table 3:** Description of clusters predicted by several clustering algorithms

Algorithms	Category	Cluster				Average Size			
		DIP	Krogan	MIPS	Gavin	DIP	Krogan	MIPS	Gavin
CMC [16]	Density	1263	907	168	486	4.39	4.56	–	5.55
COACH [17]	Core	903	547	448	361	3.89	8.97	–	8.26
MCL [19]	Flow	623	932	–	425	6.57	3.62	–	3.93
MCODE [15]	Density	63	85	85	150	19.00	5.88	–	6.63
ClusterOne [18]	Graph	372	373	256	312	4.90	4.29	–	6.35
CFinder [14]	Density	609	88	–	137	6.18	12.73	–	9.6
DBSCAN [19]	Density	492	96	130	24	6.26	34.43	14.3	12.7
OPTICS	Density	107	278	439	108	5.90	4.17	9	13.5
OPTICS_PSO [20]	Density	76	119	98	84	5.59	8.05	33	8.45
iOPTICS-GSO	Density	99	143	86	101	5.76	5.62	26.5	8.14

From Table 3, we can see that the numbers of clusters obtained by the proposed algorithm on four datasets are smaller than those compared methods. The reason of this result is that the number of interactions in most sub-networks is sparse, so the distance of these nodes calculated by Eq. (7) would be up to 1, and these nodes were regarded as a class, respectively. In the final phase, we filtered the results from each sun-network clustering, and deleted some clustering modules whose density was smaller or had only one node.

**Fig. 5:**
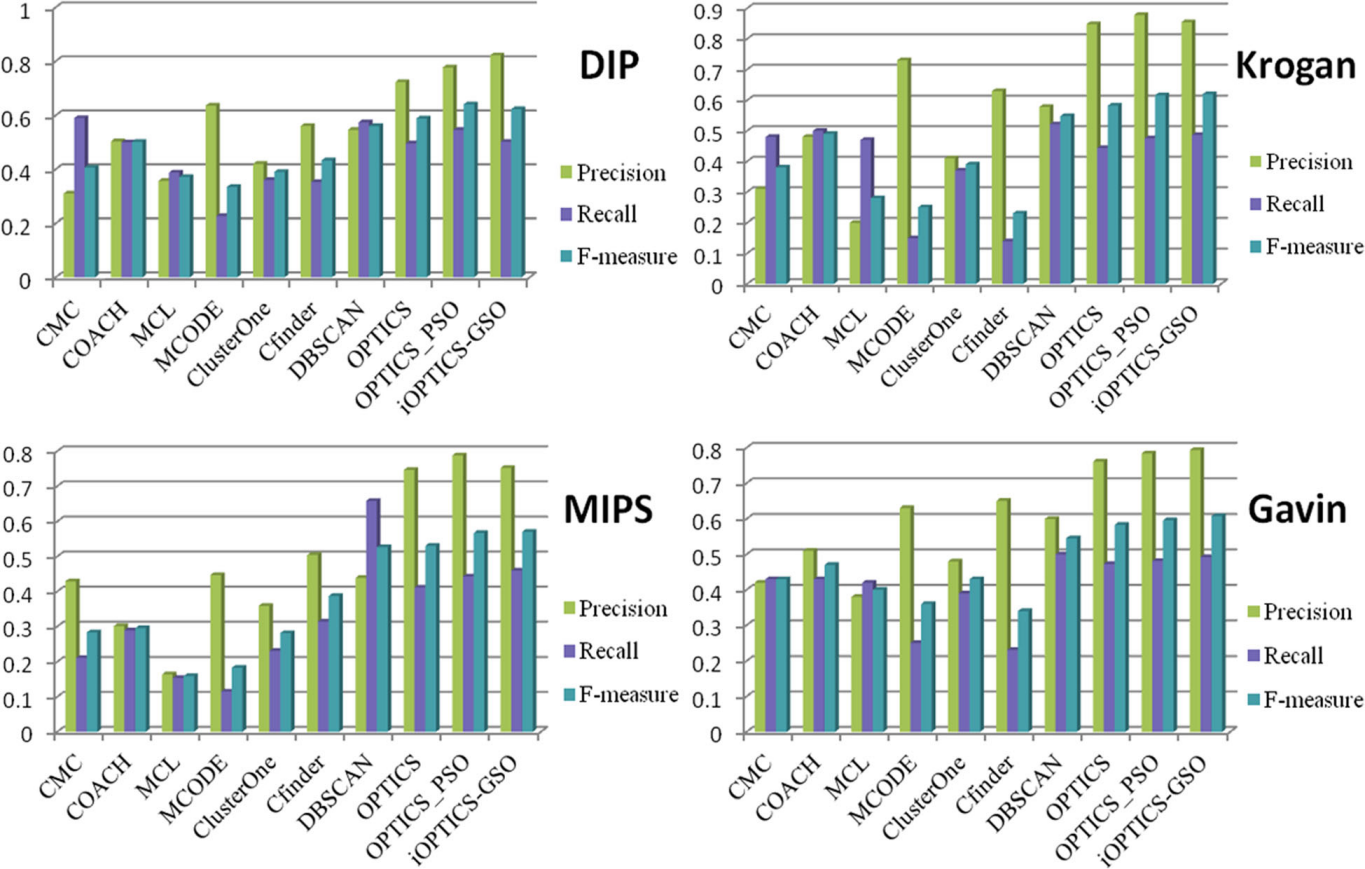
The performance comparisons of various algorithms on four datasets

Fig. 5 depicts the *precision*, *recall*, *f-measure* of each algorithm on four datasets. From Fig. 5, we can see that the proposed algorithm obtains the higher *precision* and *f-measure* than other competing algorithms. After combining OPTICS with GSO algorithm, the iOPTICS-GSO algorithm can produce the clustering results based on the optimal parameters. Therefore, it obtains a much better performance than the OPTICS algorithm. From the last green and blue column in Fig. 5, we can clearly see that the proposed algorithm obtains the higher *precision* and *f-measure* than other competing algorithms.

To evaluate the biological significance and functional enrichment of the complexes identified by our algorithm, we calculated the *p-value* of the identified protein complexes on Biological Process ontologies based on four datasets by using the tool SGD’s GO: TermFinder (http://www.yeastgenome.org/cgi-bin/GO/goTermFinder.pl). We calculate the *p-value* of the protein complexes identified by six algorithms, COACH, MCL, MCODE, ClusterOne, OPTICS and OPTICS_PSO, whose size are greater than or equal to 3. The comparison results are showed in Table 4. From Table 4, it is obvious that the proposed algorithm achieves the better performance on DIP data, Krogan data, MIPS data and Gavin data. While the MCL and ClusterOne obtain poor performance on four datasets. There is a few protein complexes identified by iOPTICS-GSO that are insignificant. Especially on the Krogan data, no protein complex is insignificant. That is to say, all protein complexes identified by iOPTICS-GSO on Krogan data are significant. In detail, in DIP data, Krogan data, and Gavin data, the percentages of complexes with *p-value* < E-15 in predicted complexes by iOPTICS-GSO was the highest. It accounted for 8.70%, 12.22% and 26.25%, respectively. In MIPS data, the percentage of complexes with *p-value* < E-15 in protein complexes identified by iOPTICS-GSO was the highest. It accounted for 20.00%. As for the comparison with OPTICS_PSO, the percentage of complexes which are significant identified by iOPTICS-GSO was the higher on DIP data and Krogan data. In MIPS data and Gavin data, the percentage of complexes with *p-value* < E-10 in protein complexes identified by iOPTICS-GSO was the higher. In general, the statistical results in Table 4 indicate that iOPTICS-GSO algorithm was more biologically meaningful than others for identifying significant protein complexes.

**Table 4:** Comparison of the functional enrichment of protein complexes with other algorithms on four datasets

Dataset	Algorithm	<E-15	[E-15, E-10]	[E-10, E-5]	[E-5, 0.01]	<0.01 significant	≥0.01 insignificant
DIP	COACH	33(6.96%)	44(9.28%)	205(43.25%)	126(26.58%)	408(86.08%)	66(13.92%)
	MCL	19(1.80%)	47(4.46%)	183(17.38%)	362(34.38%)	611(58.02%)	442(41.98%)
	MCODE	12(7.27%)	17(10.30%)	80(48.48%)	38(23.03%)	147(89.09%)	18(10.91%)
	ClusterOne	21(3.66%)	52(9.06%)	177(30.84%)	184(32.06%)	434(75.61%)	140(24.39%)
	OPTICS	7(7.87%)	13(14.61%)	40(44.94%)	21(23.6%)	81(91.01%)	8(8.99%)
	OPTICS_PSO	5(6.85%)	10(13.70%)	27(36.99%)	23(31.51%)	65(89.04%)	8(10.96%)
	**iOPTICS-GSO**	**6(8.70%)**	**15(21.74%)**	**29(42.03%)**	**13(18.84%)**	**63(91.30%)**	**6(8.70%)**
Krogan	COACH	23(10.41%)	37(16.74%)	91(41.18%)	54(24.43%)	205(92.76%)	16(7.24%)
	MCL	16(3.97%)	43(10.67%)	103(25.56%)	119(29.53%)	281(69.73%)	122(30.27%)
	MCODE	8(5.00%)	28(17.50%)	68(42.50%)	46(28.75%)	150(93.75%)	10(6.25%)
	ClusterOne	13(3.26%)	43(10.78%)	98(24.56%)	120(30.08%)	274(68.67%)	125(31.33%)
	OPTICS	13(8.44%)	26(16.88%)	56(36.36%)	31(20.13%)	126(81.82%)	28(18.18%)
	OPTICS_PSO	9(9.47%)	19(20.0%)	41(43.16%)	21(22.11%)	90(94.74%)	5(5.26%)
	**iOPTICS-GSO**	**11(12.22%)**	**23(25.56%)**	**37(41.11%)**	**19(21.11%)**	**90(100%)**	**0(0%)**
MIPS	COACH	16(4.04%)	46(11.62%)	145(36.62%)	149(37.63%)	356(89.9%)	40(10.10%)
	MCL	5(0.83%)	13(2.15%)	94(15.51%)	220(36.30%)	332(54.79%)	274(45.21%)
	MCODE	5(3.70%)	10(7.41%)	70(51.58%)	39(28.89%)	124(91.85%)	11(8.15%)
	ClusterOne	7(1.88%)	16(4.30%)	117(31.45%)	126(33.87%)	266(71.51%)	106(28.49%)
	OPTICS	16(5.63%)	6(2.11%)	26(9.15%)	74(26.06%)	122(42.96%)	162(57.04%)
	OPTICS_PSO	10(11.76%)	3(3.53%)	28(32.94%)	30(35.29%)	71(83.53%)	14(16.47%)
	**iOPTICS-GSO**	**7(11.67%)**	**5(8.33%)**	**12(20%)**	**25(41.67%)**	**49(81.67%)**	**11(18.33%)**
Gavin	COACH	35(14.96%)	39(16.67%)	100(42.72%)	55(23.50%)	229(97.86%)	5(2.14%)
	MCL	22(9.69%)	34(14.98%)	88(38.77%)	66(29.07%)	110(92.51%)	17(7.49%)
	MCODE	12(7.74%)	20(12.90%)	80(51.61%)	39(25.16%)	151(97.42%)	4(2.58%)
	ClusterOne	31(10.62%)	34(11.64%)	118(40.41%)	82(28.08%)	292(90.75%)	27(9.25%)
	OPTICS	20(18.52%)	13(12.04%)	53(49.07%)	19(17.59%)	105(97.22%)	3(2.78%)
	OPTICS_PSO	15(18.07%)	13(15.66%)	38(45.78%)	16(19.28%)	82(98.80%)	1(1.20%)
	**iOPTICS-GSO**	**21(26.25%)**	**11(13.75%)**	**31(38.75%)**	**16(20%)**	**79(98.75%)**	**1(1.25%)**

The bold data in Tables 4 are the result of our four datasets

**Fig. 6:**
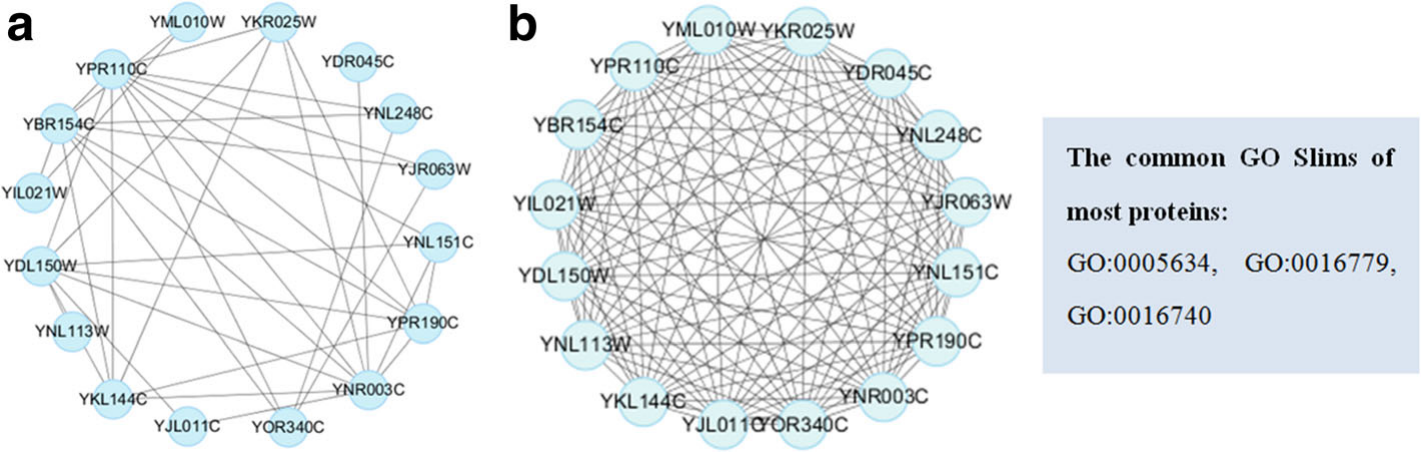
Visualization of a protein complex (ID1 in Table 5). YKL144C, YNR003C, YPR110C, YPR190C, YDL150W, YKR025W, YNL151C, YBR154C, YJL011C, YNL113W, YDR045C, YNL248C, YJR063W, YOR340C, YIL021W, YML010W are the names of proteins, which represent different proteins

We list some identified protein complexes in Gavin data shown in Table 5. These protein complexes are not well matched with the benchmark dataset (the value of *OS* is low), but both have low *p-value* of GO terms. The *p-value* of the identified protein complexes is calculated on Molecular Function. In each row, the proteins in bold have well matched some known protein complex in benchmark complex dataset, and the additional proteins probably share the similar functions with other proteins. For example, 5 proteins do not matches the known protein complex in the first predicted protein complex, while 4 proteins of which (namely YNL248C, YJR063W, YOR340C and YIL021W) share the similar annotations—DNA-directed 5′-3′ RNA polymerase activity—with the true protein complex. We visualize this protein complex shown in Fig. 6. Fig. 6a describes the interaction relationship between 16 proteins, and (b) shows the common GO slim between every two proteins. We can see clearly that the interactions in (a) are much less than those in network (b). This shows that even if there is no interaction between some proteins, but they still have the common GO slim, meaning that they as complex implement some functions with a high probability. Given the incompleteness of protein complex set, the predicted protein complexes have low value of *OS* but with small *p-value* are highly likely to be true protein complexes. Therefore, the results provided clues for biologists to verify and find new protein complexes.

**Table 5:** Some examples of the predicted complexes with small *p-value* on Gavin data

No.	Predicted protein complex	*p-value*	Gene Ontology term	*OS*
1	**YKL144C YNR003C YPR110C YPR190C YDL150W YKR025W YNL151C YBR154C YJL011C YNL113W YDR045C** YNL248C YJR063W YOR340C YIL021W YML010W	1.22E-35	DNA-directed 5'-3' RNA polymerase activity (GO:0003899)	0.44
2	**YJL069C YLR409C YLR222C YLR129W YDR449C YCR057C** YGL171W YDR365C YKR060W YDR299W YGR145W YDL213C YNL075W YHR148W YLR186W YLL011W YJR002W YPL217C YGR128C YNL132W YMR093W YCL059C YPR144C YER082C YPR137W YBR247C YPL126W YDR324C YHR196W YOR078W YDL148C YJL109C YMR128W YOL010W YNL308C YHR169W YPR112C YDL166C YLR003C YGR081C YOR056C YGR054W YKL143W YNL207W YPL204W YCL011C YJL033W YKL059C YLR115W YAL043C YLR277C YNL317W YKL018W YJR093C	5.46E-32	snoRNA binding (GO:0030515)	0.11
3	**YML114C YCR042C YPL011C YDR167W YMR236W YBR198C YGL112C YMR005W YML015C YDR145W YMR227C** YBR081C YLR055C YDR448W YGR252W YDR392W YPL254W	2.37E-26	transcription factor activity (GO:0001075)	0.47
4	**YCR042C YML114C YMR005W YML015C YPL011C YMR236W YGR274C YBR198C YGL112C** YLR055C YCL010C YDR448W YPL254W	1.67E-21	transcription factor activity (GO:0001075)	0.41
5	**YLR129W YLR409C YDR449C YCR057C** YPL266W YPR112C YDR299W YGR128C YPL126W YJR002W YDR324C YNL132W YPL217C YBL004W YDL148C YER082C YHR196W YGR090W YCL059C YLR003C YCL011C YCL031C YDL213C	2.91E-17	snoRNA binding (GO:0030515)	0.12
6	**YLR418C YGL244W YOL145C YBR279W YOR123C** YGL019W YOR039W YMR309C YPL181W	6.89E-14	RNA polymerase II C-terminal domain phosphoserine binding (GO:1990269)	0.36
7	**YHL025W YBR289W YPL016W YPR034W YJL176C YFL049W YHR023W** YPL082C YNL059C YNL272C YML114C YPL011C YDR176W YBR198C YDR392W YGL066W YOL148C YDR145W YER164W YKR001C YDR073W YML069W YKL088W YMR172W	4.3E-11	DNA-dependent ATPase activity (GO:0008094)	0.17
8	**YHR156C YHR165C YER172C YPR082C** YDL087C YGR013W YDR283C YJL203W YDR416 YGL128C YLR117C YAL032C YPR178W YBL104C YGL100W YIL061C	2.45E-07	second spliceosomal transesterification activity (GO:0000386)	0.07

The proteins in bold have well matched some known protein complex in benchmark complex dataset

## Conclusions

Protein complexes are not only the basis of normal biological processes, but also play an important role in the pathological process. Therefore, identifying protein complexes play an important role in understanding the cellular organizations and functional mechanisms. In this study, we have put forward the algorithm named iOPTICS-GSO, which is the improved OPTICS algorithm by using GSO to optimize the parameter in OPTICS, and we changed the concept of core node and redefine the similarity which makes more accord with the actual situation of PPI network. As different parameter setting have different results on each sub-network of DPIN, we have used GSO algorithm to optimize these parameters, and finally checked the quality of every cluster and gained the optimal cluster results. The experiment results have shown that our iOPTICS-GSO outperforms competing algorithms in terms of *f-measure* and *p-value*. It means the results from iOPTICS-GSO are more biologically meaningful than others for identifying significant proteins complexes. However we also found that the number of clustering modules is relatively small and the recall of clustering results is lower than other algorithms in iOPTICS-GSO results. The reason may be that each protein only can belong to one cluster in iOPTICS-GSO, which causes that other clustering modules are small. Therefore, it would be our focus to discover the effective strategy to improve the result and detect more protein complexes in the future.
